# The association of breastfeeding duration with later maternal feeding styles in infancy and toddlerhood: a cross-sectional analysis

**DOI:** 10.1186/1479-5868-10-53

**Published:** 2013-04-26

**Authors:** Katherine Isselmann DiSantis, Eric A Hodges, Jennifer Orlet Fisher

**Affiliations:** 1 Department of Community & Global Public Health, Arcadia University, College of Health Sciences, 450 S. Easton Road, 219 Brubaker Hall, Glenside, PA, 19038-3295, USA; 2 The University of North Carolina at Chapel Hill, School of Nursing, Chapel Hill, NC, USA; 3 Department of Public Health, Temple University, College of Health Professions and Social Work, Philadelphia, PA, USA

**Keywords:** Breastfeeding, Satiety response, Feeding behaviors, Feeding responsiveness

## Abstract

**Background:**

Breastfeeding modestly reduces obesity risk, yet the mechanisms are not well understood. The goal of the current research was to evaluate the association of breastfeeding duration with a wide range of maternal feeding approaches in late infancy and toddlerhood.

**Methods:**

A secondary analysis of cross-sectional data from an ethnically-diverse sample of 154 mothers of infants (aged 7–11 months) and toddlers (aged 12–24 months) was performed. Breastfeeding history was self-reported where 75% of mothers had weaned by the time of the interview. Multiple dimensions of maternal feeding approaches were measured using the Infant Feeding Styles Questionnaire which assesses pressuring, restriction, responsive, laissez-faire, and indulgent approaches to feeding. Analyses were performed separately for infants and toddlers and adjusted for maternal education level, ethnicity, and marital status.

**Results:**

Mothers of infants who breastfed for longer durations tended to report greater responsiveness to infant satiety cues (p≤0.01) and reduced pressuring in feeding complementary foods (p<0.05). Mothers of toddlers who breastfed for longer durations tended to report reduced pressuring in feeding complementary foods (p<0.01).

**Conclusion:**

These results suggest that breastfeeding may shape maternal feeding approaches related to responsiveness to infant cues as infants enter a period of complementary feeding, even after considering a range of demographic characteristics previously associated with breastfeeding behaviors. That responsiveness to feeding cues was not associated with breastfeeding duration in the toddler sample suggests that some aspects of this association might be isolated to infancy.

## Background

A modest protective effect of breastfeeding on the development of obesity from childhood into adolescence and adulthood is well established [1-3]. A recent study using a sibling model found breastfed siblings were about 13 pounds lighter than non-breastfed siblings (based on same height) in early adolescence [4]. The association of breastfeeding with obesity risk appears to be dose-dependent, supporting a causal relationship [2,5]. However, concerns over whether this evidence is a result of publication bias and confounding have led some experts to say the modest, positive effect of breastfeeding on obesity might not exist [6]. Despite these concerns, the most recent policy statement on breastfeeding from the American Academy of Pediatrics included obesity prevention as one of the numerous benefits of breastfeeding, citing a 24% reduction in obesity risk [7].

Potential mechanisms of the breastfeeding-obesity relationship have been explored [8], but remain poorly understood. The "hows" or behavioral aspects of breastfeeding are considered to be an important dimension [9]. The infant-centered nature of breastfeeding is thought to influence the amount of control mothers impose during feeding, entraining high levels of maternal sensitivity to infant feeding cues. Highly controlling approaches to feeding have been suggested to have detrimental influence on child regulation of appetite by being potentially unresponsive to child hunger and satiety cues [10,11], and are often assessed by measuring restrictive and pressuring approaches to feeding. Highly restrictive approaches to feeding, for instance, are thought to distract children from hunger/satiety cues by focusing attention on the availability of restricted foods [12], whereas highly pressuring approaches are thought to encourage eating in the absence of hunger or past fullness [12]. Prospective findings have provided some support for the contention that breastfeeding might entrain lower levels of maternal control [13-15], showing associations between longer breastfeeding duration and lower subsequent maternal restriction of infant feeding at 6- [13,15] and 12-months of age [14]. In turn, maternal control of infant feeding has been identified as a mediating factor between breastfeeding and energy intake in toddlerhood [16]. A study of 1012 mother-infant pairs found that maternal restriction during infant feeding (measured when infant was 12 months) partially attenuated the inverse relationship between breastfeeding duration and later body mass index [BMI] z-score [14]. These findings establish that breastfeeding is associated with less maternal control in the first 6–12 months of feeding. Less clear is whether the experience of breastfeeding shapes maternal control in feeding later in infancy and beyond, particularly as the child moves into the period of complementary feeding. Toddlerhood brings more complexity in caregiver-infant feeding interactions with a movement towards self-feeding and adult-like meal patterns. Thus, understanding whether the experience of breastfeeding shapes subsequent approaches to and sensitivity in child feeding holds interest.

Beyond expanding inquiry into the developmental stage of toddlerhood, widening the evaluation of maternal feeding beyond highly controlling approaches is important [16-18]. While maternal control has been a focal aspect of maternal feeding approaches in the study of child overweight and obesity [16-18], recent work has attempted to better understand the broader range of feeding approaches among ethnically diverse caregivers of children from birth to 5 years of age [19,20]. General and feeding-specific styles of parenting reflect the broad emotional climate in which specific goal-directed practices are used and in which socialization takes place [19]. Styles consider overall demands and expectations placed on the child as well as the caregiver’s responsiveness to the child’s unique needs [21]. Authoritative parenting, which balances demands on and responsiveness to the child, has been related to lower BMI in preschoolers and older children [19,22]. Conversely, indulgent approaches to general parenting and feeding, which involves high responsiveness to the child with few demands, has been related to higher BMI and less nutrient-dense food intake in preschoolers [19,23,24]. Potential associations of breastfeeding with authoritative or indulgent feeding approaches and practices have not been well investigated, yet the evidence for older children encourages the evaluation of a broader range of feeding styles earlier in life. Additionally, because ethnic differences in both rates of child overweight and obesity [25] and in parent feeding styles [19,26,27] have been identified, studies of maternal feeding are most informative when performed in diverse samples.

This investigation addresses these gaps in the literature by evaluating the association of breastfeeding duration with a wide range of maternal feeding styles later in infancy and toddlerhood including pressuring, restrictive, responsiveness, indulgence, and laissez-faire feeding styles [20] in an ethnically diverse sample. It was hypothesized that mothers with higher breastfeeding duration levels would report higher responsiveness, less restriction, and less pressuring in feeding their infant or toddler. Given relationships between indulgent feeding and overweight in older children [19,23], it was hypothesized that longer breastfeeding durations would be related to lower amounts of indulgent feeding in infancy and toddlerhood.

## Methods

A secondary analysis was performed using data from a cross-sectional study of dietary assessment methodology among ethnically diverse infants and toddlers [21]; the methods of the study have been previously described [21]. Briefly, convenience sampling was used to recruit mother-child dyads, including recruiting through a volunteer database maintained at the US Department of Agriculture (USDA) Children’s Nutrition Research Center and recruiting in-person and/or posting fliers at targeted locations (including childcare classes, festival, doctors’ offices, retail stores, and churches). Data were collected in 2005 and 2006. Reflecting the design of the study, children in the dyads were either infants aged 7–11 months (n=79) or toddlers aged 12–24 months (n=74). Potential participants were also intentionally sampled to include equal representation of Hispanics, blacks, and whites. Maternal inclusion criteria were self-identification in one of these race/ethnicity groups and having primary responsibility for infant feeding at home. Infant inclusion criteria were being within the study age range at the time of enrollment, born at full-term (37–42 wk), and birth weight-for-age of >5th percentile [28]. Infant exclusion criteria were significant feeding problems which results in a highly restrictive diet (e.g. severe food allergies, severe reflux), chronic medical conditions, and/or medication use; maternal reports of such problems were considered by the research team on a case-by-case basis.

### Measures

#### Family demographic and maternal characteristics

Demographic information was collected by maternal self-report including maternal race/ethnicity, maternal education level, parity at birth of the child participant, marital status, and household income level. Mothers also reported whether their family participated in a US governmental food and nutrition assistance program (Women, Infants and Children [WIC]). Maternal height (Harpenden tadiometer; Holtain Limited) and weight [Doctor Scale 431/432KL series (capacity: 400_0.25 lb or 181.4 _ 0.1 kg); Health-O-Meter, Bridgeview IL] were measured in duplicate by trained research staff according to the methods of Lohman et al. [29], where repeat measurements were performed in cases of non-agreement (within 0.5 kg or 0.5 cm). BMI scores were calculated based on these measures.

#### Breastfeeding and infant feeding history

Breastfeeding duration was self-reported by the mother in months. Mothers were asked “Has your child ever been breastfed or fed breastmilk?”. If mothers responded “yes”, they were asked “Are you currently breastfeeding?”. For mothers who were currently breastfeeding at the time of interview, their infant’s age in months at the time of interview was recorded as the duration of “any breastfeeding”. Last, if mothers were not currently breastfeeding, they were asked “How old was your child when you stopped breastfeeding?”. Duration was categorized in three categories based on US public health recommendations and population trends [7]. While current US recommendations endorse exclusive breastfeeding for 6 months [7], national breastfeeding rates show marked shifts between birth and 3 months, and again from 3 to 6 months; the percent of mothers breastfeeding drops from 75% at birth to 35% at 3 months, and to 14% at 6 months [30]. Therefore, three categories of breastfeeding duration corresponding to those recommendations and trends were utilized: 0–2.99 months [BF<3], 3–6 months [BF3-6], > 6 months [BF>6]. Previously cited dose-dependent relationships between breastfeeding and obesity [2,5] also support the use of a graded classification of duration. To determine age of formula supplementation, mothers were asked, “How old was your baby when you started feeding formula?” (If mothers answered “No” to the question “Has your child ever been fed formula?”, this question was skipped). To determine age at introduction of complementary foods, mothers were asked at what age (in months) their infant was first fed the following baby foods: cereal, fruit, vegetables, meats, and mixed dishes. The lowest age was recorded as the age at introduction of complementary foods.

#### Feeding styles

The Infant Feeding Styles Questionnaire [IFSQ] was utilized to assess maternal feeding styles reflecting a broad range of maternal beliefs and behaviors surrounding infant and toddler feeding [20]. Styles were informed by Costanzo and Woody’s [31] theory of domain-specific parenting styles, where maternal feeding styles are based on concerns (e.g. concern over eating unhealthy foods, concern about child weight status) and constraints (e.g. resources, knowledge). The IFSQ includes items that assess feeding styles in five domains: (1) Laissez-faire, (2) Pressuring/controlling, (3) Restrictive/controlling, (4) Responsive, and (5) Indulgent. Each domain contains multiple subscales for a total of 13 subscales with 83 items; exemplars are presented in Table 1. Laissez-faire feeding was measured with two subscales, *Diet Quality* and *Attention,* and higher scores represent fewer caregiver limits and less interaction. Pressuring/controlling feeding was measured with three subscales, *Finishing Cereal, and Soothing.* Higher scores on *Finishing* subscale represent greater caregiver pressure on infant to finish a bottle/food during a meal; higher scores on the *Cereal* subscale relate to offering cereal in a bottle to their infant and to beliefs that cereal consumption before the age of six months is needed for infant to sleep and feel full; and high scores on the *Soothing* subscale represent greater caregiver pressure to eat in an effort stop an infant’s/toddler’s crying. Restrictive/controlling feeding was measured with two subscales, *Amount* and *Diet Quality*, and higher scores represent more caregiver limits on the quantity and quality (in terms of healthfulness) of food their infant/toddler consumes. Responsive feeding was measured with two subscales, *Satiety and Hunger Cue* and *Attention/Interactions*. Higher scores on the *Satiety and Hunger Cues subscale* represent greater caregiver responsiveness to infant/toddler hunger and satiety cues and a greater belief in infant/toddler ability to self-regulate. Higher scores on the *Attention/Interactions subscale* represent more general attentiveness during feedings. Indulgent feeding was measured with three subscales, *Coaxing, Pampering*, and *Soothing*, and higher scores represent less caregiver limit setting on the quantity or quality of food consumed with greater feeding to coax, soothe, or pamper the infant/toddler. Feeding behavior items were scored from “1”-never to “5”-always and feeding beliefs were scored from “1”-disagree to “5”-agree. The behavior portion of the Laissez-faire *Diet Quality* Subscale (4 questions) was scored in descending order where “1”-always to “5”-never.

**Table 1:** Example items from IFSQ by subscale

**Style Domains**	**Sub-Scale Name**
**Responsive**	**Satiety (7 items)**
Behavior	*(Child) lets me know when s/he is full*
Belief	*Child knows when s/he is full*
**Responsive**	**Attention (5 items)**
Behavior	*Talk to (child) to encourage him/her to eat*
Belief	*Important to help or encourage a toddler to eat*
**Restrictive**	**Amount (4 items)**
Behavior	*I carefully control how much (child) eats*
Belief	*Important parent decides how much infant should eat*
**Restrictive**	**Diet Quality (7 items)**
Behavior	*I let (child) eat fast food*
Belief	*A toddler should never eat fast food*
**Pressuring**	**Finishing (8 items)**
Behavior	*Try to get (child) to finish his/her food*
Belief	*Important for toddler finish all food on his/her plate*
**Pressuring**	**Cereal (5 items)**
Behavior	*Give/gave (child) cereal in the bottle*
Belief	*An infant <6 mo needs more than formula or breastmilk to be full*
**Pressuring**	**Soothing (4 items)**
Behavior	*When (child) cries, I immediately feed him/her*
Belief	*Best way to make infant stop crying is to feed*
**Indulgent**	**Coaxing (8 items)**
Behavior	*Allow child to eat desserts/sweets to make sure s/he gets enough*
Belief	*Toddlers should be allowed to eat desserts/sweets to make sure they get enough*
**Indulgent**	**Soothing (8 items)**
Behavior	*Allow child watch tv while eating to keep him/her from crying*
Belief	*Toddlers should be allowed to watch tv while eating to keep them from crying*
**Indulgent**	**Pampering (8 items)**
Behavior	Allow child to eat desserts/sweets to keep him/her happy
Belief	Toddlers should be allowed to eat desserts/sweets to keep them happy
**Laissez-faire**	**Attention (5 items)**
Behavior	*When (name of child) has/had a bottle, I prop/propped it up*
Belief	*I think it is okay to prop an infant’s bottle*
**Laissez-faire**	**Diet Quality (6 items)**
Behavior	*I keep track of what food (child) eats†*
Belief	*A toddler should be able to eat whatever s/he wants for snacks*

†This question was reverse coded.

In previous studies, the internal consistency of sub-scale items ranged from moderate to strong and all but three of the subscales had desirable reliability (H coefficients ≥0.80) in a sample of 154 3–20 month old low-income, African American infants/toddlers [20]. The remaining three subscales (restriction in amount of food, pressuring to eat cereal, pressuring to finish meal) had satisfactory internal consistency (H coefficients ≥0.75) [20]. Evidence of predictive validity is demonstrated by associations of four of the thirteen constructs with infant weight-for-length z-score [20]. In the current study, the internal consistency of the IFSQ subscales was assessed for the entire sample. All subscales under the Indulgent and Pressuring domains were good or acceptable (α≥0.7). For the Restrictive subscales, the internal consistency of the Restrictive *Diet Quality* subscale was good after removing two questions intended for mothers of toddlers-only (α≥0.7). For the Responsive scales, both the *Satiety* and *Attention* subscales had questionable internal consistency (α≥0.6); the internal consistency of the *Satiety* scale was acceptable when evaluated in the infant sample alone (α=0.7). The internal consistency of the Laissez-faire *Diet Quality* subscale was acceptable (α=0.7), but the Laissez-faire *Attention* subscale had questionable internal consistency (α<0.6).

#### Infant or toddler anthrometrics

Anthropometrics included length and weight measured in triplicate by trained research staff. Electronic scales [3862 MP 6; Sartorius (readability: 0.1 g)] were used to measure weight and infant length boards (Holtain Limited, Crymych, United Kingdom) were used to measure length of both infants and toddlers. The 2000 Centers for Disease Control and Prevention growth charts were used to calculate weight-for-length z scores (referred to as relative weight) [32].

### Statistical analyses

Descriptive variables (family demographic, maternal characteristics, infant relative weight (weight-for-length z-score)) and infant feeding variables (breastfeeding duration, supplementation, solid introduction) were examined for the infant and toddler samples separately, and compared between the three breastfeeding groups (BF<3, BF3-6, BF>6), using ANOVA and Chi-square analyses as appropriate. Potential maternal covariates included education (college degree or more vs. less than college degree) [30], race/ethnicity [30], income [30], age [30], marital status (married vs. not) [30], and maternal weight status (overweight/obese vs. not) [33]. Potential child covariates included relative weight [34] and gender [35] which have been previously associated with breastfeeding and/or parent feeding styles. Potential covariates were included in the ANCOVA models based on significant associations (p≤0.05) with the IFSQ subscales, based on ANOVA analyses and Spearman correlation as appropriate, and were considered separately for the infants and toddler groups. All significant associations are presented in Tables 2 and 3 for the infant and toddler samples, respectively. If a potential covariate was significantly associated with a subscale from more than one of the five feeding domains, it was included as a covariate. Neither of the potential child covariates (relative weight, gender) was significantly associated with any of the subscales in either the infant or toddler samples; thus, they were not included in ANCOVA models. Based on those preliminary analyses, maternal education, marital status, and maternal ethnicity were included as potential covariates in the ANCOVA models for both infants and toddler samples. Bonferroni post hoc tests were used to understand differences in effects between groups.

**Table 2:** Associations identified between potential covariates and IFSQ subscales in the infant sample

	**Responsive subscales**	**Restrictive subscales**	**Pressuring to eat subscales**	**Indulgent subscales**	**Laissez-Faire subscales**
Infant Gender					
Infant Relative Weight^a^					
Maternal Education Level	Amount of Food Consumed**		Cereal**	Coaxing**	Attention**
			Finishing Food*	Pampering*	
	Diet Quality**			Permissive**	
				Soothe*	
Maternal Ethnicity	Amount of Food Consumed**		Cereal**	Coaxing**	Attention**
			Finishing Food**	Pampering*	
			To Soothe**	Permissive**	
				Soothe*	
Family Income level			Cereal*		
			Finishing Food*		
Marital Status	Diet Quality*	Satiety and Hunger Cues**	Cereal**	Coaxing**	Attention**
				Pampering*	
				Permissive**	
				Soothe*	
Maternal Weight Status			Cereal*		

*p<0.05

**p<0.01

^a^ The association between infant relative weight and the IFSQ subscales were assessed by Spearman Correlation, due to the continuous nature of the covariate, while Analysis of Variance analyses were performed for all other covariates.

Note: All categorical covariates were coded as follows: Gender (Male=1, Female=2); Maternal Education Level (College Grad or Higher=1, Less than College Grad=0); Maternal Ethnicity (White=1, Black=2, 3=Hispanic); Income level (1=less than $35,000, 2=$35,000-49,999, 3=$50,000-74,999, 4=$75,000-99,999, 5=100,000 or greater); Marital Status (Married=1, Not Married=0); Maternal weight status (Overweight/Obese=1, Not overweight/obese=0).

**Table 3:** Associations identified between potential covariates and IFSQ subscales in the toddler sample

	**Responsive subscales**	**Restrictive subscales**	**Pressuring to eat subscales**	**Indulgent subscales**	**Laissez-Faire subscales**
Infant Gender					
Infant Relative Weight^a^					
Maternal Education Level	Diet Quality**	Satiety and Hunger Cues*	Cereal**	Coaxing*	
				Permissive*	
				Soothe*	
Maternal Ethnicity			Cereal**	Coaxing*	Attention*
			To Soothe*	Soothe*	
Family Income Level				Pampering*	
Marital Status	Diet Quality*			Coaxing**	Attention*
	Amount of Food Consumed*		Cereal**	Permissive**	
				Soothe**	
Maternal Weight Status					

*p<0.05

**p<0.01

^a^ The association between infant relative weight and the IFSQ subscales were assessed by Spearman Correlation, due to the continuous nature of the covariate, while Analysis of Variance analyses were performed for all other covariates.

Note: All categorical covariates were coded as follows: Gender (Male=1, Female=2); Maternal Education Level (College Grad or Higher=1, Less than College Grad=0); Maternal Ethnicity (White=1, Black=2, 3=Hispanic); Income level (1=less than $35,000, 2=$35,000-49,999, 3=$50,000-74,999, 4=$75,000-99,999, 5=100,000 or greater); Marital Status (Married=1, Not Married=0); Maternal weight status (Overweight/Obese=1, Not overweight/obese=0).

## Results & discussion

### Sample

Tables 4 and 5 present the demographic characteristics of mothers of infants and toddlers, respectively. A high proportion of mothers were employed and college graduates. Mothers of infants and toddlers who breastfed longer (BF>6 ) were more likely to be college educated and less likely to participate in WIC (p<0.05) compared to mothers who breastfed for shorter durations (BF<3, BF3-6). For mothers of infants only, mothers who breastfed longer (BF>6) were less likely to report current employment (p<0.05) compared to mothers who breastfed for shorter durations (BF<3, BF3-6). For mothers of toddlers only, mothers who breastfed longer (BF>6) were more likely to be non-Hispanic white (p<0.05) compared to mothers who breastfed for shorter durations (BF<3, BF3-6). There were no significant differences in relative weight between breastfeeding duration groups in either the infant or toddler samples.

**Table 4:** Demographics of infant sample by breastfeeding groups

**Demographics**	**All infants**	**BF< 3mos**	**BF 3-6mos**	**BF>6mos**	**p-value**
	**n=79**	**n=21**	**n=28**	**n=30**	
Mother Age, mean (SD)	30.4 (5.4)	28.9 (5.9)	30.0 (5.3)	31.8 (5.0)	0.16
Months of Any Breastfeeding, mean (SD)**	5.5 (3.6)	0.8 (0.8)	4.3 (1.2)	9.5 (1.5)	0.00
Months of Exclusive Breastfeeding, mean (SD)	2.4 (2.2)	0.5 (0.8)	1.8 (1.7)	3.6 (2.3)	0.00
Age at Introduction of Complementary foods (Months), mean (SD)	4.3 (1.3)	3.6 (1.1)	3.8 (1.5)	4.9 (1.1)	0.00
Infant Weight-for-length z-score	−0.02 (0.9)	−0.03 (1.0)	0.20 (0.8)	0.14 (0.9)	0.14
Never put Cereal in Bottle?†	45.9%	33.3%	32.1%	72.0%	0.01
Infant Gender					0.36
Male	46.3%	33.3%	53.6%	48.4%	
Female	53.8%	66.7%	46.4%	51.6%	
Mom Race Ethnicity					0.06
White, non-Hispanic	37.5%	19%	32.1%	54.8%	
Black, African American	30%	42.9%	25%	25.8%	
Hispanic	32.5%	38.1%	42.9%	19.4%	
Parity					0.07
Primiparious at Child’s Birth	45%	66.7%	35.7%	38.7%	
Household Income Level					0.61
< $35,000	21.8%	35%	21.4%	13.3%	
$35,000-45,999	23.1%	25%	21.4%	23.3%	
$50,000-$74,999	20.5%	15%	25%	20%	
$75,000-$99,999	16.7%	10%	21.4%	16.7%	
≥$100,000	17.9%	15%	10.7%	26.7%	
Maternal Education Level*					0.01
College Graduate?	50%	42.9%	32.1%	71.0%	
Mother Employed?*	58.8%	76.2%	71.4%	35.5%	0.01
Mom Overweight or Obese	72.6%	85.0%	69.6%	66.7%	0.34
Family Participates in WIC*	40%	47.6%	57.1%	19.4%	0.01

*p<0.05.

**p<0.01.

^a^For mothers who were still breastfeeding, the duration of breastfeeding was put as their infant’s age at the time of enrollment.

^b^Based on answering “Never” to the question, “I give/gave my child cereal in the bottle.”

**Table 5:** Demographics of toddler sample and by breastfeeding groups

**Demographics**	**All toddlers**	**BF< 3mos**	**BF 3-6mos**	**BF>6mos**	**p-value**
	**n=75**	**n=21**	**n=14**	**n=40**	
Mother Age, mean (SD)	30.5 (5.5)	30.8 (7.3)	27.9 (4.5)	31.2 (4.6)	0.15
Months of Any Breastfeeding, mean (SD)**	7.9 (6.5)	0.4 (0.5)	4.9 (1.0)	12.7 (4.8)	0.00
Months of Exclusive Breastfeeding, mean (SD)	3.0 (2.8)	0.2 (0.3)	1.7 (1.5)	4.5 (2.0)	0.01
Age at Introduction of Complementary Foods (Months), mean (SD)	4.9 (1.5)	4.2 (1.4)	4.4 (1.6)	5.2 (1.5)	0.00
Toddler Weight-for-length z-score	−0.13 (1.0)	−0.10 (1.1)	0.36 (0.9)	0.32 (0.9)	0.08
Never put Cereal in Bottle?†	45.9%	47.4%	30.8%	83.3%	0.01
Infant Gender					0.71
Male	42.1%	38.1%	35.7%	46.3%	
Female	57.9%	61.9%	64.3%	53.7%	
Mom Race Ethnicity*					0.03
White, non-Hispanic	32.9%	14.3%	21.4%	46.3%	
Black, African American	31.6%	47.6%	50%	17.1%	
Hispanic	35.5%	38.1%	28.6%	36.6%	
Parity					0.93
Primiparious at Child’s Birth	52.6%	52.4%	57.1%	51.2%	
Household Income Level					0.24
< $35,000	28.3%	28.6%	50%	15.0%	
$35,000-45,999	18.7%	23.8%	7.1%	20.0%	
$50,000-$74,999	20.0%	25.8%	7.1%	22.5%	
$75,000-$99,999	14.7%	9.4%	7.1%	20.0%	
≥$100,000	21.3%	14.3%	28.6%	22.5%	
Maternal Education Level*					0.02
College Graduate?	61.8%	38.1%	57.1%	75.6%	
Mother Employed?	57.9%	52.4%	57.1%	61.0%	0.81
Mom Overweight or Obese	52.9%	47.4%	63.6%	29.4%	0.69
Family Participates in WIC**	21.1%	38.1%	42.9%	4.9%	0.00

*p<0.05

**p<0.01

^a^For mothers who were still breastfeeding, the duration of breastfeeding was put as their infant’s age at the time of enrollment.

^b^Based on answering “Never” to the question, “I give/gave my child cereal in the bottle.”

### Breastfeeding and infant feeding history

Table 4, presents mean breastfeeding and exclusive breastfeeding (only human milk) duration for mothers of infants across the three groups. Twenty-seven mothers (34.1%) were still breastfeeding (non-exclusively) at the time of the interview. There were significant differences in age at introduction of complementary foods (p<0.01), with post-hoc analyses showing that infants in the BF<3 group (*M*=3.6 months, *SD*=1.1) and the BF3-6 months group (*M*=3.8 months, *SD*=1.7) received complementary foods earlier than the BF>6 group (*M*=4.9 months, *SD*=1.1). Table 5 presents mean breastfeeding and exclusive breastfeeding lengths for mothers of toddlers across the three groups. Twelve mothers (16.0%) were still breastfeeding (non-exclusive) at the time of the interview. There were significant differences in age at introduction of complementary foods (p<0.01), with post-hoc analyses finding that toddlers in the BF<3 group (*M*=4.2 months, *SD*=1.4) and the BF3-6 group (*M*=4.4 months, *SD*=1.6) received complementary foods earlier than the BF>6 group (*M*=5.2 months, *SD*=1.5).

### Breastfeeding duration and maternal feeding styles

**Figure 1:**
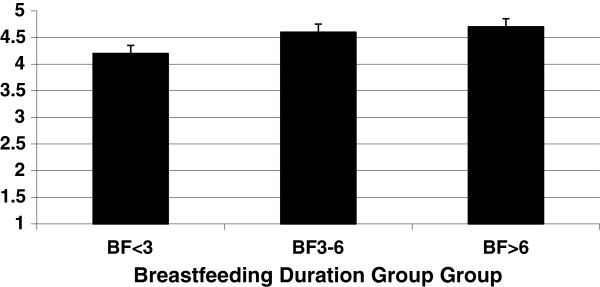
**Breastfeeding duration group group.** Maternal responsiveness to satiety of infants (n=79) by breastfeeding duration group. Bars show mean score on the subscale. ANCOVA revealed a significant difference between the BF<3 and the BF3‒6 groups (p<0.05) and the BF<3 and the BF>6 groups (p<0.05).

Mothers of infants who breastfed longer reported greater responsiveness to infant satiety cues (p≤0.01) and less pressuring of their infant (related to infant cereal consumption) (p≤0.01) (Table 6). Post-hoc analyses are presented in Figure 1 and show for the responsiveness to satiety cues subscale that the mothers in the BF>6 (*M*=4.7, *SD*=0.4) and the BF3-6 groups (*M*=4.6, *SD*=0.4) reported higher responsiveness (p<0.05) compared to the BF<3 group (*M*=4.2, *SD*=0.7). The BF3-6 and BF>6 groups did not differ (p>0.05) in the amount of responsiveness reported by mothers of infants. Post-hoc analyses for the pressuring to eat cereal subscale showed that the mothers in the BF>6 group (*M*=1.6, *SD*=0.8) reported exerting less pressure to eat cereal (p<0.01) compared to the BF3-6 (*M*=2.8, *SD*=1.0) and BF<3 (*M*=2.6, *SD*=1.1) groups. The BF3-6 and BF<3 groups did not differ (p>0.05) in the amount of pressure reported by mothers of infants. There were no significant differences (p>0.05) between the breastfeeding duration groups for any of the subscale scores under the indulgent, restrictive, or laissez-faire feeding styles.

**Table 6:** ANCOVA† of Breastfeeding duration as a function of maternal feeding styles among infants

**IFSQ Subscale**	**BF<3 Mon (n=21)**	**BF 3–6 Mon (n=28)**	**BF>6 Mon (n=30)**	**p-value**
**Responsiveness Subscales**				
Satiety and Hunger Cues*	4.2 (0.7)	4.6 (0.4)	4.7 (0.4)	0.02
Attention & Interactions	3.4 (0.7)	3.3 (0.7)	3.5 (0.7)	0.65
**Restriction Subscales**				
Amount of Food Consumed	3.3 (0.9)	3.2 (1.0)	2.8 (1.1)	0.37
Diet Quality	2.8 (0.6)	2.8 (0.6)	3.0 (0.6)	0.92
**Pressuring to Eat Subscales**				
To Soothe	2.4 (0.9)	2.0 (0.7)	2.1 (0.8)	0.11
Finishing Food	2.4 (0.7)	2.4 (0.6)	2.1 (0.8)	0.80
Cereal*	2.6 (1.1)	2.8 (1.0)	1.6 (0.8)	0.00
**Indulgent Subscales**				
Coaxing	1.4 (0.5)	1.3 (0.3)	1.3 (0.4)	0.62
Pampering	1.4 (0.5)	1.3 (0.3)	1.2 (0.4)	0.86
Permissive	1.7 (0.7)	1.8 (0.6)	1.7 (0.6)	0.38
Soothing	1.4 (0.5)	1.1 (0.2)	1.2 (0.3)	0.32
**Laissez-faire Subscales**				
Attention	1.8 (0.7)	1.99 (0.7)	1.8 (0.6)	0.25
Diet Quality	2.2 (0.8)	2.40 (1.0)	2.1 (0.7)	0.55

†Covariates included were maternal ethnicity, marital status, and maternal education level.

*p<0.05

Note: IFSQ Subscales were scored from 1–5, where behaviors are scored from “1”-never to “5”-always and beliefs are scored from “1”-disagree to “5”-agree. The behavior portion of the Laissez-Faire Diet Quality Subscale was scored in descending order where “1”-always to “5”-never. IFSQ=Infant Feeding Styles Questionnaire; BF<3 Mon=Breastfed less than 3 months group; BF3-6 Mon= Breastfed for 3–6 months group; BF>6 Mon= Breastfed for more than 6 months group.

Mothers of toddlers who breastfed longer reported lower pressuring in relation to infant cereal consumption (p≤0.01) (Table 7). Post-hoc analyses showed that the BF>6 group (*M*=1.4, *SD*=0.5) reported exerting less pressure to eat cereal (p<0.01) compared to the BF3-6 group (*M*=2.4, *SD*=0.8) and BF<3 group (*M*=2.6, *SD*=1.2). The BF3-6 and BF<3 groups did not differ in the amount of pressure reported by mothers. It should be noted that the questions regarding pressuring to eat cereal center on the introduction of complementary foods to infants and focus on the first six months of life. For the BF<3 group and BF3-6 groups, 86.3% and 78.6% of mothers of toddlers reported introducing cereal prior to the age of six months, respectively, compared to 57.5% for mothers of toddlers in the BF>6 group. There were no significant differences (p>0.05) between the breastfeeding duration groups for any of the subscale scores under the responsive, indulgent, restrictive, or laissez-faire feeding styles.

**Table 7:** ANCOVA† of Breastfeeding Duration as a Function of Maternal Feeding Styles among Toddlers

**IFSQ Subscale**	**BF<3 Mon (n=21)**	**BF 3–6 Mon (n=14)**	**BF>6 Mon (n=41)**	**P-value**
**Responsiveness Subscales**				
Satiety and Hunger Cues	4.6 (0.3)	4.5 (0.4)	4.4 (0.4)	0.15
Attention & Interactions	3.8 (0.7)	3.7 (0.7)	3.7 (0.7)	0.84
**Restriction Subscales**				
Amount of Food Consumed	3.2 (1.1)	3.1 (1.1)	2.8 (0.9)	0.18
Diet Quality	2.8 (0.5)	3.0 (0.5)	3.0 (0.5)	0.34
**Pressuring to Eat Subscales**				
To Soothe	2.3 (0.8)	2.1 (0.6)	2.3 (0.8)	0.72
Finishing Food	2.6 (0.9)	2.5 (0.8)	2.3 (0.7)	0.56
Cereal*	2.6 (1.2)	2.4 (0.8)	1.4 (0.5)	0.00
**Indulgent Subscales**				
Coaxing	1.7 (0.7)	1.4(0.4)	1.4(0.4)	0.28
Pampering	1.6 (0.9)	1.5 (0.4)	1.4 (0.4)	0.85
Permissive	2.1 (1.0)	2.0 (0.8)	1.9 (0.5)	0.86
Soothing	1.4 (0.7)	1.4 (0.4)	1.3 (0.4)	0.95
**Laissez-faire Subscales**				
Attention	2.1 (0.7)	2.1 (0.7)	1.8 (0.6)	0.20
Diet Quality	2.1 (0.8)	2.3 (0.6)	2.4 (0.9)	0.57

†Covariates included were maternal ethnicity marital status and maternal education level.

*p<0.05.

** p<0.01.

Note: IFSQ Subscales were scored from 1–5, where behaviors are scored from “1”-never to “5”-always and beliefs are scored from “1”-disagree to “5”-agree. The behavior portion of the Laissez-Faire Diet Quality Subscale was scored in descending order where “1”-always to “5”-never. IFSQ=Infant Feeding Styles Questionnaire; BF<3 Mon=Breastfed less than 3 months group; BF3-6 Mon= Breastfed for 3–6 months group; BF>6 Mon= Breastfed for more than 6 months group.

## Discussion

This research studied breastfeeding and its relationship with a wide range of feeding styles during infancy and toddlerhood in an effort to advance scientific understanding of potential behavioral factors that underlie the protective effect of breastfeeding on child obesity risk. The main finding was that mothers who breastfed 3 months or longer reported higher levels of responsiveness to infant satiety and hunger cues than mothers who breastfed for less than 3 months, after taking into consideration potentially important covariates (maternal education, marital status, and ethnicity). These results suggest that mothers who breastfeed may show greater acknowledgement of infants’ ability to communicate fullness and responsiveness to those cues during infant feeding. This association was not observed among mothers of toddlers, suggesting that the relationship may be limited to infancy.

Our findings suggest that responsiveness is at the core of feeding style differences between mothers of varying breastfeeding duration, which augments past research on the positive relationship between breastfeeding and child-centered which focused on control [8,36-38]. It is plausible that mothers who breastfed for a longer duration differ in attitudes and beliefs which influence level of responsiveness/pressuring feeding styles in a manner unexplained by the socio-demographic characteristics taken into account in this study. However, it could be that the more a mother engages in a behavior, the more likely she is to permanently adopt the behavior. In this case, longer breastfeeding duration would allow a mother to engage in infant-centered feeding interactions for a longer time, increasing the likelihood that these interaction patterns would continue, after initiation of complementary feeding [39]. This possibility suggests that the infant-centered nature of breastfeeding, if of sufficient duration, results in greater responsiveness to infant feeding cues, which is consistent with prior research findings in this area [13,15,16]. While Brown and Lee [38] found a shorter duration of breastfeeding (6 weeks) was sufficient to predict satiety responsiveness among toddlers regardless of current feeding style, they did not report relationships between duration and feeding style. It is also interesting that a difference in maternal responsiveness was identified between mothers breastfeeding for less than three months and those breastfeeding for 3 or more months. Currently only 35% of mothers in the US are breastfeeding exclusively at three months [21], which suggests greater supports need to be put in place to extend duration beyond 3 months in order to maximize the benefits of breastfeeding in regards to responsive feeding.

Despite the inclusion of multiple dimensions of feeding styles in the current study, breastfeeding duration was largely unassociated with indulgent, restrictive, laissez-faire, and pressuring maternal feeding styles. Breastfeeding duration was associated with less maternal pressure among both infants and toddlers, but only in relation to early introduction of infant cereal. Thus mothers who breastfed for greater than 6 months were less likely to offer cereal in the bottle and/or to hold beliefs that the use of infant cereal in early infancy (<6 months) offers benefits (mainly infant fullness and sleeping through the night). Past research on pressuring has primarily relied on the Child Feeding Questionnaire, which focuses on pressuring a child to finish a meal, to eat without hunger, and to eat “enough” from the caregiver’s perspective [17]. The measure used here dimensionalized pressuring feeding styles *(pressure to finish, pressure to soothe, and pressure to eat supplementary foods early in infancy).* That only aspects of pressuring related to complementary food introduction were associated with breastfeeding duration suggests dimensionalizing pressuring feeding styles offers more insight to their connection with breastfeeding duration. While responsiveness to satiety and hunger cues has been consistently linked to reduced obesity risk [20,40,41], there is less consistent evidence that early introduction of complementary foods increases obesity risk [42-46], particularly among breast-fed infants [47,48].

Few associations of breastfeeding duration with feeding styles were observed among mothers of toddlers. It is possible that greater length of recall by mothers of toddlers could have increased the greater chance of recall bias and misclassification relative to mothers of infants. However, maternal self-reports of breastfeeding are considered to be fairly reliable in the first three years of life [49]. It is also possible that the lack of association reflects a dynamic nature of feeding behaviors and interactions in toddlerhood. During toddlerhood, a number of intrinsic factors (within the developing child) are transacting with numerous extrinsic factors, including multiple caregivers responsible for feeding [50], modeling influences of siblings and peers [51], and the child’s increasing involvement in family meals, which are often determined by the family’s social clock rather than its individual members’ hunger cues. Because the design of this study was not longitudinal, causal inferences are not appropriate; however the findings encourage longitudinal inquiry. Longitudinal research that spans late infancy into toddlerhood would offer more insights on whether breastfeeding duration in early infancy continue to influence maternal responsiveness during feeding, particularly considering the increasing intrinsic and extrinsic factors described here.

This study had a number of strengths and limitations. While ethnically diverse, this sample of mothers was generally college educated, of middle income, and married. Also, mothers in this sample had higher rates of overweight/obesity (72.6%) than in the US adult population (64.1%) [36]. However, weight was recorded in the postpartum period which likely inflated the rates for this sample. Results might be different in samples with more diversity in terms of these maternal characteristics, particularly for education level due to its positive association with breastfeeding duration [52]. This study was not able to ascertain the extent to which breastfeeding duration involved feeding at the breast, referred to as “direct breastfeeding”, versus feeding human milk in a bottle. Direct breastfeeding has been linked to improved infant self-regulation of formula/human milk intake [49] and later satiety response [37], as well as reduced risk of accelerated weight gain [53]; thus future research in this area should differentiate between direct breastfeeding and feeding human milk in a bottle. Last, relative weight was included only as a potential covariate here. Future studies investigating breastfeeding behaviors and feeding styles would benefit from a longitudinal design which can assess relative weight at multiple time points into early childhood, particularly in larger samples which might identify even modest associations between breastfeeding behaviors, feeding styles, and child weight measures.

## Conclusions

This research provides new evidence that longer breastfeeding duration may increase mothers’ responsiveness to infant feeding cues after initiation of complementary feeding. It is clear that the first two years are highly important for obesity prevention [54] and that breastfeeding plays a role in prevention [55]. These findings suggest that breastfeeding might offer protection via responsive feeding during infancy, but that effects on maternal feeding beliefs and practices may not directly carry-over into toddlerhood. Further study is required to understand why breastfeeding might impact feeding styles and how that impact is related to obesity risk. If the behavioral factors of breastfeeding which lead mothers to engage in less obesogenic feeding behaviors during infancy are better understood, then promotion of healthier feeding behaviors could be extended to all caregivers during infancy, toddlerhood, and beyond, regardless of breastfeeding duration.

### Consent

Written informed consent was obtained from the participant's parent/caregiver for the publication of this report.

## Abbreviations

USDA: US Department of Agriculture; BF<3: Breastfeeding duration of 0–2.99 months; BF3-6: Breastfeeding duration of 3–6 months; BF>6: Breastfeeding duration of > 6 months; IFSQ: Infant feeding styles questionnaire; BMI: Body mass index.

## Competing interests

The authors declare that we have no competing interests.

## Authors’ contribution

Significant writer (KID, EAH), significant reviewer (EAH and JOF), manuscript concept/design (KID), data acquisition (JOF), and data analysis (KID). All authors have read and approved the final manuscript.
